# Supplementary material for: Identification of photoperiod-induced specific miRNAs in the adrenal glands of Sunite sheep (Ovis aries)
Source: Front Vet Sci. 2022 Jul 22;9:888207. doi: 10.3389/fvets.2022.888207 (PMC9354845; doi:10.3389/fvets.2022.888207)
Supplement: Supplementary file 1 [file Data_Sheet_1.ZIP › Supplementary file/miRDeep2 file.zip!/miRDeep2 file/Novel_Structure/LP42A2/Novel_322.pdf]

Provisional ID	:	NC_019484.2_37784
Score total	:	2.5
Score for star read(s)	:	-1.3
Score for read counts	:	0
Score for mfe	:	2.2
Score for randfold	:	1.6
Score for cons. seed	:	
Total read count	:	57
Mature read count	:	57
Loop read count	:	0
Star read count	:	0

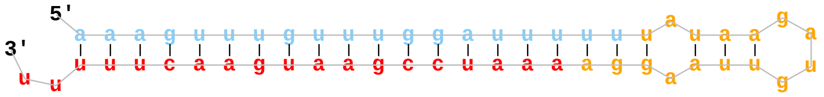

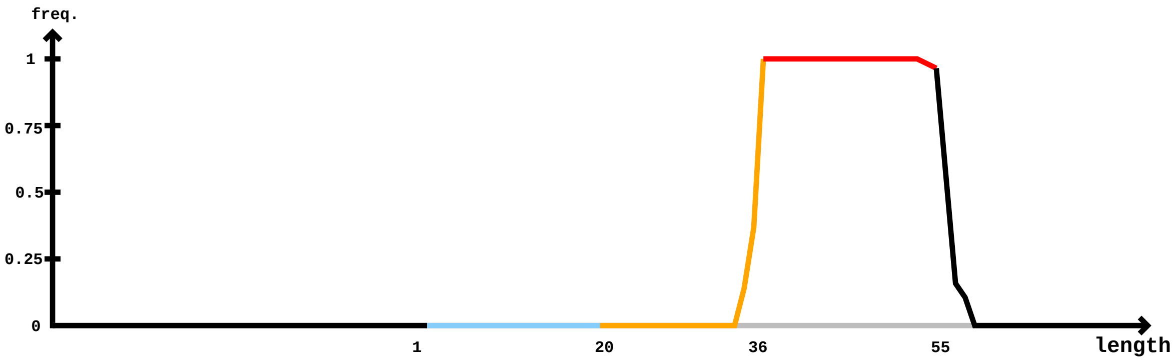


```
                                     Star                           Mature
5'- uacaugugcgaauuguuacuaauauugggcuggccaaaaaguuuguuuggauuuuuuauaagauguuaaggaaaauccgaaugaacuuuuuggccugcccaauacaucugcc   -3'   exp
    ......................((((((((.((((((((((((((((((((((((((.(((....))).)))))))))))))))))))))))))).)))))))).......   reads   mm   sample
    ......................................................................gaaaauccgaaugaacuuu.......................   1       0    seq
    ......................................................................gaaaaCccgaaugaacuuuu......................   1       1    seq
    ......................................................................gaaaauccgaaugaacuuuuu.....................   2       0    seq
    ......................................................................gaaaaucUgaaugaacuuuuug....................   1       1    seq
    ......................................................................gaaaauccgaaugaacuuuuug....................   3       0    seq
    .......................................................................aaaauccgaaugaacuuuuu.....................   8       0    seq
    .......................................................................aaaaCccgaaugaacuuuuu.....................   1       1    seq
    .......................................................................aaaaucAgaaugaacuuuuug....................   1       1    seq
    .......................................................................aaaaCccgaaugaacuuuuugg...................   2       1    seq
    .......................................................................aaaauccgaaugaacuuuuugg...................   1       0    seq
    ........................................................................aaaCccgaaugaacuuuuu.....................   28      1    seq
    ........................................................................aaauccgaaugaacuuuuu.....................   1       0    seq
    ........................................................................aaauccgaaugaacuuuuug....................   1       0    seq
    ........................................................................aaaCccgaaugaacuuuuuggc..................   6       1    seq
```
